# Estimation of the Excess COVID-19 Cases in Seoul, South Korea by the Students Arriving from China

**DOI:** 10.3390/ijerph17093113

**Published:** 2020-04-29

**Authors:** Sukhyun Ryu, Sheikh Taslim Ali, Jun-Sik Lim, Byung Chul Chun

**Affiliations:** 1 Department of Preventive Medicine, College of Medicine, Konyang University, Daejeon 35365, Korea; gentryu@onehealth.or.kr; 2 Korean Society of Epidemiology 2019-nCoV Task Force Team, Korea; 3 WHO Collaborating Centre for Infectious Disease Epidemiology and Control, School of Public Health, Li Ka Shing Faculty of Medicine, The University of Hong Kong, Hong Kong, China; alist15@hku.hk; 4 College of Veterinary Medicine and Institute of Veterinary Science, Kangwon National University, Chuncheon 24341, Korea; borizook@onehealth.or.kr; 5 Department of Preventive Medicine, Korea University College of Medicine, Seoul 02841, Korea

**Keywords:** coronavirus, COVID-19, simulation, quarantine, compliance, Korea

## Abstract

*Background*: In March 2020, overall, 37,000 international students from China, a country at risk of the 2019-novel coronavirus (COVID-19) infection has arrived in Seoul, South Korea. Individuals from the country at risk of COVID-19 infection have been included in the Korean home-quarantine program, but the efficacy of the program is uncertain. *Methods*: To estimate the possible number of infected individuals within the large influx of international students from China, we used a deterministic compartmental model for epidemic and performed a simulation-based search of different rates of compliance with home-quarantine. *Results*: Under the home-quarantine program, the number of the infected individuals would reach 40–72 from 12 March–24 March with the arrival of 0.2% of pre-infectious individuals. Furthermore, the number of isolated individuals would peak at 40–64 from 13 March–27 March in Seoul, South Korea. Our findings indicated when incoming international students showed strict compliance with quarantine, epidemics by the international student from China were less likely to occur in Seoul, South Korea. *Conclusions*: To mitigate possible epidemics, additional efforts to improve the compliance of home-quarantine of the individuals from countries with the virus risk are warranted along with other containment policies.

## 1. Introduction

Three major respiratory virus-related events have been observed in South Korea in the 21st century: Severe acute respiratory virus (SARS), Middle East respiratory syndrome, and the 2019-novel coronavirus (COVID-19) infection, all of which are caused by members of the coronavirus family. The first individual with COVID-19 infection in South Korea was identified on 20 January, 2020, and the number of laboratory-confirmed cases increased between then and 12 February, 2020 [1]. To reduce the number of individuals entering South Korea who may have been exposed to COVID-19 in Wuhan, China, an international travel ban from Hubei Province, China to South Korea was implemented on 3 February 2020 [2]. Furthermore, to identify individuals who may have been exposed to COVID-19, the South Korean public health authority implemented a quarantine program. Any persons who have travelled from a country with COVID-19 infection risk within the previous 14 days or have been in contact with laboratory-confirmed COVID-19 infection within the previous 14 days is defined as an individual for quarantine [3]. Quarantined individuals are asked to comply with home-quarantine and are monitored by local public health workers twice a day for 14 days after contact with individuals with infection [3].

On 14 February, 2020, the South Korean public health authority identified an individual with COVID-19 infection; the patient had been contacted by another individual who was suspected of avoiding the quarantine program during his period of home-quarantine [4]. According to previous literature, the effectiveness of quarantine varies widely depending on individuals’ daily motility patterns [5]; Despite this compliance with home-quarantine in the present instance is still in question.

It is important to note that 37,000 students from China, where major cities were experienced localized outbreaks on February 2020 [6], has entered Seoul, South Korea, on 1 March, 2020 at the start of the spring semester. This large number of incoming youths from the country with COVID-19 infection risk may increase the risk of local transmission in South Korea.

In this study, we aimed to estimate the number of infected and isolated individuals, expected in Seoul, South Korea, based on compliance with home-quarantine and proportion of pre-infectious individuals among these incoming international students from China, a country at risk for COVID-19 infection.

## 2. Materials and Methods

**Figure 1:**
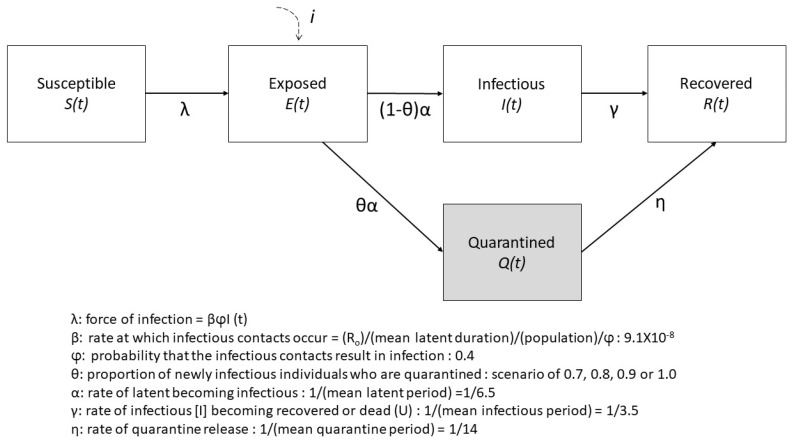
Graphical illustration of deterministic SEIR compartment model with quarantine. The population is divided into five classes: susceptible, exposed (infected with the virus but not infectious yet), infectious (symptomatic only), and recover. Exposed individuals are quarantined on contact tracing with a fraction of the compliance rate.

To simulate possible epidemics, we used the deterministic compartmental model of susceptible–exposed–infectious–removed type. The population is divided into five distinct classes: Susceptible, exposed (infected with the virus but not symptomatic), quarantined (with a fraction of compliance rate), infectious (and symptomatic), and removed (or recovered from the infection). The rate of change at time *t* in the number of susceptible (*S*(*t*)), exposed (*E*(*t*)), infectious (*I*(*t*)), removed (*R*(*t*)) and quarantine (*Q*(*t*)) under the model specification as illustrated in Figure 1, are summarized by a set of differential equations in the Supplementary Material.

Where the parameters including *β*, *φ*, *α*, *γ*, *θ*, and η were the rate at which infectious contact occur, probability that the infectious contact result in successful infection, rate of exposed individuals becoming infectious, rate of infectious individuals becoming recovered, proportion of newly exposed individuals were quarantined, and rate of quarantine release, respectively. The basic reproductive number ($R_{0}$) and the rate at which infectious contact occur were assumed to be 2.68, and 9.1 × 10^−8^ based on the $R_{0}$, *α*, *φ*, and the population size in Seoul (9.74 million) [6,7,8,9]. We assumed the model parameters as: *φ* = 0.4 [8], *α*^−1^ = 6.5 days [7], γ^−^^1^ = 3.5 days [10], and η^−^^1^ = 0.07. The additional pre-infection seeding (*i*) as the international students from China would be influx into the exposed class.

We assumed that the population mixed homogeneously, and that no COVID-19 transmission had occurred within the community in Seoul. Furthermore, we assumed that either 0.1%, 0.2%, or 1% of the incoming international students (*i*) were in the pre-infectious period of COVID-19 infection, based on previous literature reporting that 0.2% of individuals with contactees of SARS infection were asymptomatic [11]. We also assumed that the international students would arrive in Seoul, South Korea in the 15 days before and after 1 March, 2020, and that no individuals were isolated during entry screening upon arrival. Furthermore, we assumed that all quarantined individuals were confined at home or to the university dormitory as per the current South Korean quarantine program for COVID-19. The baseline scenarios were based on the currently identified number of infected persons from China in South Korea, which was 12 on 6 February, 2020 [1], with the assumption of 90% compliance with home-quarantine during the pre-infectious period. Scenarios with different quarantine compliance rates (70%, 80%, 90%, or 100%) among these international students as well as local cases were also modeled. We considered a time horizon of 180 days for the number of individuals infected and quarantined since 20 January, 2020, when the first COVID-19 case was identified in South Korea.

## 3. Results

**Figure 2:**
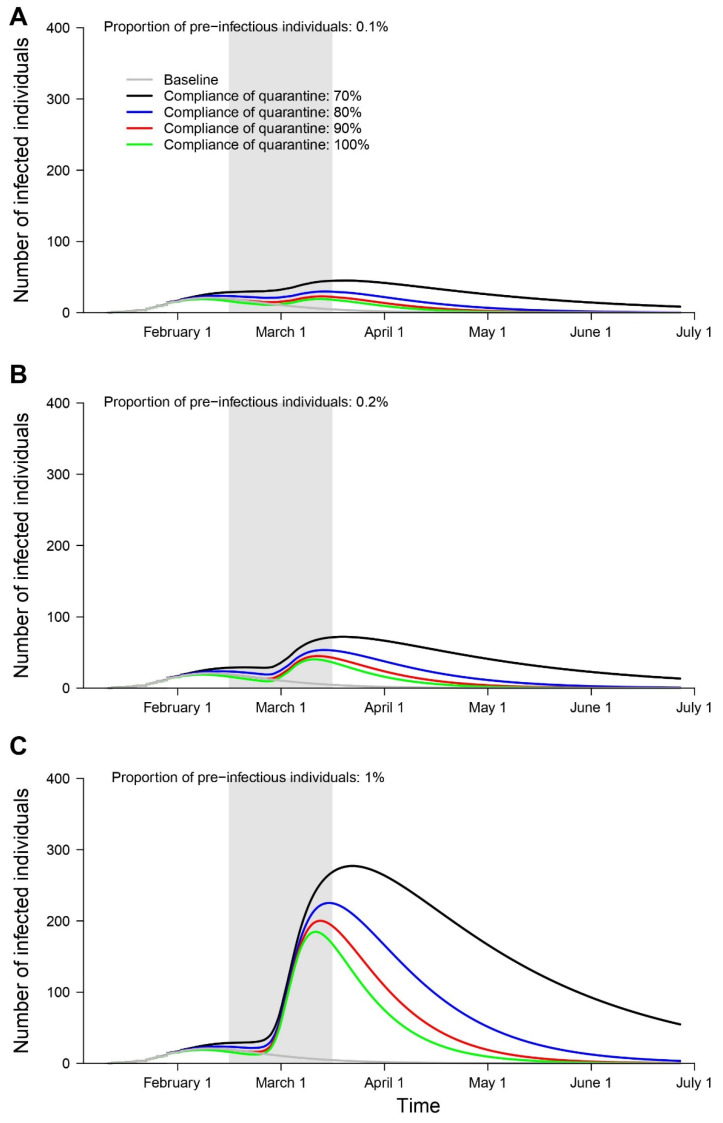
Estimated daily number of individuals with infection in Seoul, South Korea under different scenarios regarding the proportion of pre-infectious individuals: 0.1% (**A**), 0.2% (**B**), and 1% (**C**), based on different compliance rates with home-quarantine (gray: baseline, black: 70%, blue: 80%, red: 90%, green: 100%). Shaded gray bars represent the period of arrival of the international students from China, the country with the risk of 2019 novel coronavirus on February 2020.

We estimated that the total number of infected individuals would reach 19–45 from March 13–24 March, 40–72 from 12 March–24 March, and 184–277 from 13 March–26 March with the arrival of 0.1%, 0.2%, and 1% of pre-infectious individuals, in Seoul, South Korea, respectively (Figure 2). 

**Figure 3:**
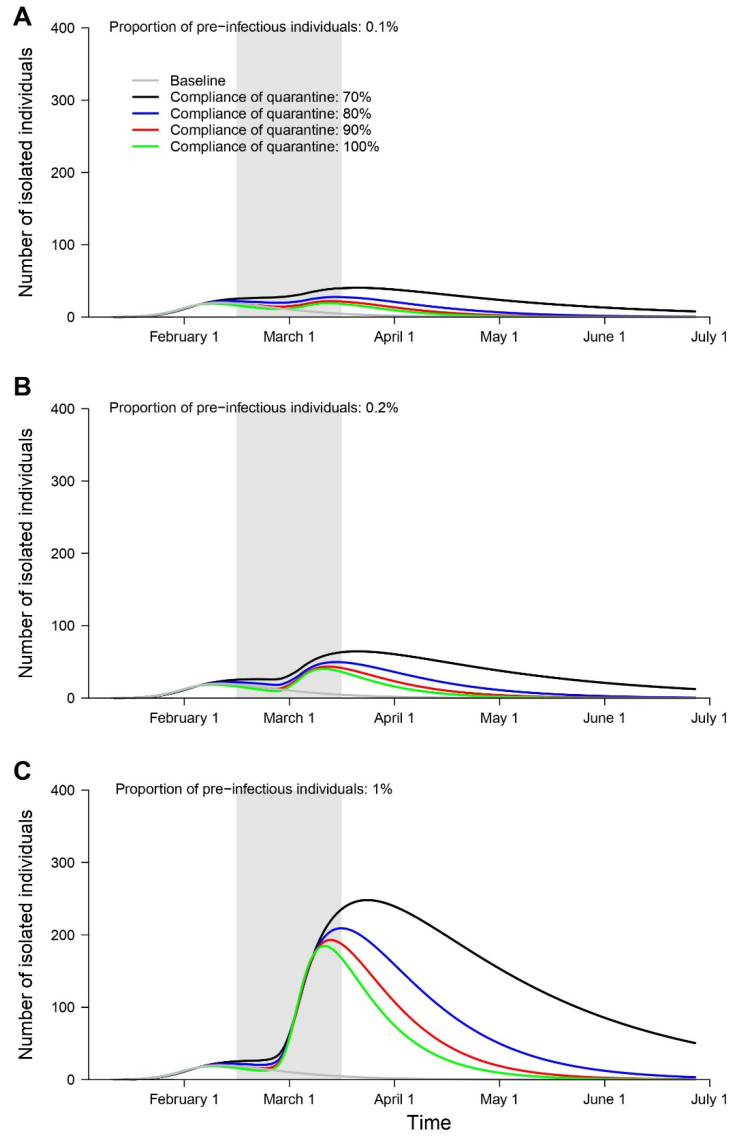
Estimated daily number of isolated individuals in Seoul, South Korea, under different scenarios regarding the proportion of pre-infectious individuals: 0.1% (**A**), 0.2% (**B**), or 1% (**C**) based on different compliance rates with home-quarantine (gray: baseline, black: 70%, blue: 80%, red: 90%, green: 100%). Shaded gray bars represent the period of arrival of the international students from China, the country with the risk of 2019 novel coronavirus on February 2020.

We also estimated that the number of individuals isolated from the South Korean quarantine program would peak at 19–40 from 13 March–28 March, 40–64 from 13 March–27 March, and 184–248 from 13 March–27 March with the arrival of 0.1%, 0.2%, and 1% of pre-infectious individuals in Seoul, South Korea, respectively (Figure 3). The number of infected and isolated individuals would increase with higher proportions of subclinical COVID-19 cases. However, the number of infected and isolated individuals was smaller due to the higher compliance of the quarantine program.

## 4. Discussion

When no effective vaccine or treatment is available for infectious disease, the quarantine of individuals suspected of having the infection, including those exposed to infection from epidemic countries, has been used as a mitigation strategy by public health authorities [12,13,14,15,16].

The number of laboratory-confirmed individuals with COVID-19 infection has been increased in China and other Asian countries. In South Korea, the likelihood of local transmission remains increasing by high because travelers are arriving from COVID-19-affected countries.

The quarantine of individuals who may have been exposed to COVID-19 is an efficient public health strategy, to reducing transmission while using limited public health resources, because the presence of individuals with unidentified infection is highly likely among individuals exposed to the infectious diseases [13,17]. Therefore, the number of individuals with infection can be estimated based on compliance with home-quarantine to provide relevant evidence for public health authorities and to improve international students’ compliance with the quarantine program in advance.

In South Korea, individuals who had contacted a person with infection were asked to comply with home-quarantine and were monitored by local public health workers twice a day for 14 days post-contact [3]. Individuals who were not included in the quarantine program but had experienced any possible contact were encouraged to notify public health authorities and submit to quarantine. All daily necessities were provided to all quarantined individuals by the public health authorities to avoid possible contact with any susceptible population, as indicated by the South Korean law. Therefore, the current quarantine program in South Korea is very broad and includes a large number of people. However, to relieve the pressure on public health resources, the quarantine program for incoming international students has been monitored by the education authority [18]. This may affect the efficacy of quarantine and increase the number of infected and isolated individuals. 

Our findings indicate that the number of infected and isolated individuals could increase by mid or late March 2020. Furthermore, the quarantine program may consume a large number of public health resources because it involves monitoring quarantined individuals and isolating infected individuals. However, our findings also suggested that most of the infected individuals could be isolated from the home-quarantine program under the compliance rate of home-quarantine between 70% and 100%. Therefore, epidemics by incoming international students from China are unlikely to occur in Seoul, South Korea. 

The present study has several limitations. First of all, some parameters including the latent period and rate of infection among those in contact with a person with infection were obtained from the modelling studies of COVID-19 [6,7,10], and consequently may revise the results. Second, we used a deterministic model, and can’t evaluate the uncertainty of these estimates, which is an inherent feature and missed under current analysis. However, allowing a search of different plausible values of these parameters through this model simulation approach, ensures the reliable parameter estimates and able to mimic the future dynamics of the number of infected individuals, which is much smaller than the total population [19]. Third, for model simplicity, we did not consider transmission that occurred before symptom onset which often being undetected and might have substantial proportion of community infection in some case.

## 5. Conclusions

As public health resources are limited, quarantine of individuals who may have been exposed to infectious disease is crucial for preventing local transmission. Therefore, strict home-quarantine of individuals from countries at risk for COVID-19 infection is important to reduce the number of infected individuals and to prevent possible epidemics in the community. Additional studies measuring the compliance of self-quarantine would be valuable for the evidence based public health policy as well as modelling studies in future epidemics.

## Supplementary Materials

The following are available online at https://www.mdpi.com/1660-4601/17/9/3113/s1. The differential equations for the deterministic SEIR model used in this study.
