# Supplementary material for: Estimation of the Excess COVID-19 Cases in Seoul, South Korea by the Students Arriving from China
Source: Int J Environ Res Public Health. 2020 Apr 29;17(9):3113. doi: 10.3390/ijerph17093113 (PMC7246702; doi:10.3390/ijerph17093113)
Supplement: Supplementary file 1 [file ijerph-17-03113-s001.pdf]

## Supplementary Materials

A simple SEIR deterministic model was applied for the simulation of 2019 Novel Coronavirus (COVID-19) in the population under our study [1]. Since the study period is relatively short, we assumed the effects of demographic change on the disease dynamics could be neglected. Furthermore, we assumed there were no natural births, or deaths occurreda. Quarantine is the intervention interrupt transmission by removing infectious individuals into quarantine [2]; Therefore, individuals were each assigned to one of the states including Susceptible (S), Exposed (E), Quarantine (Q), Infectious (I) or Recovered (R).

Susceptible individuals may contract the COVID-19 with a given rate when in contact with an infectious individual, and enter the exposed state when they are in the subclinically infected but not infectious state. The transmissibility in the study expressed by using the basic reproduction number ($R_0$) which represents the mean number of secondary infections per primary infected individuals [2]. The infectious individuals transmit COVID-19 to susceptible individuals at a rate dependent on $R_0$, the probability that the infectious contact result in infection (φ). These exposed and subclinical individuals become infectious at a rate α, with $\alpha^{-1}$ representing the mean latent period of COVID-19, or quarantined at the rate of θ, which representing the compliance of quarantine. Infectious individuals are able to spread the COVID-19 during the infectious period, with representing the mean infectious duration is equal to $v^{-1}$. After this quarantine or infectious period, they enter the recovered period, acquiring immunity to COVID-19 at the rate of $v^{-1}$ or at the rate of quarantine release (the mean quarantine period, $\eta^{-1}$). The following parameters were assumed for the mean reproductive number ($R_0$), the probability that the infectious contact result in infection (φ), the duration of the mean latent period ($\alpha^{-1}$), mean infectious period ($\gamma^{-1}$), and mean quarantine period ($\eta^{-1}$): $R_0$ = 2.68 [3], φ = 0.4 [4], $\alpha^{1}$ = 6.5 days [5], $\gamma^{-1}$ = 3.5 days [6], and $\eta^{-1}$ = 0.07. The overall population size of Seoul is 9.74 million [7]. In the scenarios, the international students who are pre-infectious periods would be influx into the Exposed state. Different percentages of exposed individuals of COVID-19 and efficacy of quarantine of exposed individuals from China, a country at risk of COVID-19, were assumed. (i.e. 0.1, 0.2, or 1% of incoming international students was in the exposed state of COVID-19 with the compliance of the qurantine of those exposed individuals was 70, 80, 90% or 100%).

The difference equations for the model are summarized as following:

$$\frac{dS(t)}{dt} = -\beta\varphi I(t)S(t)$$

$$\frac{dE(t)}{dt} = \beta\varphi I(t)S(t) - (1-\theta)\alpha E(t) - \theta\alpha E(t)$$

$$\frac{dI(t)}{dt} = (1-\theta)\alpha E(t) - \gamma I(t)$$

$$\frac{dR(t)}{dt} = \gamma I(t) \ + \eta Q(t)$$

$$\frac{dQ(t)}{dt} = \theta\alpha E(t) - \eta Q(t)$$

**References**


1. Stehle, J.; Voirin, N.; Barrat, A.; Cattuto, C.; Colizza, V.; Isella, L.; Regis, C.; Pinton, J. F.; Khanafer, N.; Van den Broeck, W.; Vanhems, P., Simulation of an SEIR infectious disease model on the dynamic contact network of conference attendees. *BMC Med* **2011,** 9, 87.
2. Gani, R.; Leach, S., Transmission potential of smallpox in contemporary populations. *Nature* **2001,** 414, (6865), 748-751.
3. Wu, J. T.; Leung, K.; Leung, G. M., Nowcasting and forecasting the potential domestic and international spread of the 2019-nCoV outbreak originating in Wuhan, China: a modelling study. *The Lancet*, 395, (10225), 689-697.
4. Andradottir, S.; Chiu, W.; Goldsman, D.; Lee, M. L.; Tsui, K. L.; Sander, B.; Fisman, D. N.; Nizam, A., Reactive strategies for containing developing outbreaks of pandemic influenza. *BMC Public Health* **2011,** 11 Suppl 1, S1.
5. Backer, J. A.; Klinkenberg, D.; Wallinga, J., Incubation period of 2019 novel coronavirus (2019-nCoV) infections among travellers from Wuhan, China, 20–28 January 2020. *Eurosurveillance* **2020,** 25, (5), 2000062.
6. Danon, L.; Brooks-Pollock, E.; Bailey, M.; Keeling, M. J., A spatial model of CoVID-19 transmission in England and Wales: early spread and peak timing. *medRxiv* **2020**; published online February 14, 2020. DOI:2020.02.12.20022566 (preprint).
7. Korean Statistical Information Service Population of Korea. http://kosis.kr/index/index.do (accessed on February 6, 2020),
